# Cysteine is a limiting factor for glioma proliferation and survival

**DOI:** 10.1002/1878-0261.13148

**Published:** 2022-01-06

**Authors:** Victor Ruiz‐Rodado, Tyrone Dowdy, Adrian Lita, Tamalee Kramp, Meili Zhang, Jinkyu Jung, Ana Dios‐Esponera, Lumin Zhang, Christel C. Herold‐Mende, Kevin Camphausen, Mark R. Gilbert, Mioara Larion

**Affiliations:** ^1^ Neuro‐Oncology Branch Center for Cancer Research National Cancer Institute National Institutes of Health Bethesda MD USA; ^2^ Radiation Oncology Branch Center for Cancer Research National Institutes of Health Bethesda MD USA; ^3^ Fred Hutchinson Cancer Research Center Seattle WA USA; ^4^ Division of Neurosurgical Research Department of Neurosurgery University Hospital Heidelberg Germany

**Keywords:** cysteine, diet, glioma, glutathione, metabolism

## Abstract

Nutritional intervention is becoming more prevalent as adjuvant therapy for many cancers in view of the tumor dependence on external sources for some nutrients. However, little is known about the mechanisms that make cancer cells require certain nutrients from the microenvironment. Herein, we report the dependence of glioma cells on exogenous cysteine/cystine, despite this amino acid being nonessential. Using several ^13^C‐tracers and analysis of cystathionine synthase and cystathioninase levels, we revealed that glioma cells were not able to support glutathione synthesis through the transsulfuration pathway, which allows methionine to be converted to cysteine in cysteine/cystine‐deprived conditions. Therefore, we explored the nutritional deprivation in a mouse model of glioma. Animals subjected to a cysteine/cystine‐free diet survived longer, although this increase did not attain statistical significance, with concomitant reductions in plasma glutathione and cysteine levels. At the end point, however, tumors displayed the ability to synthesize glutathione, even though higher levels of oxidative stress were detected. We observed a compensation from the nutritional intervention revealed as the recovery of cysteine‐related metabolite levels in plasma. Our study highlights a time window where cysteine deprivation can be exploited for additional therapeutic strategies.

## Graphical abstract

Herein, we demonstrate that glioma cells rely on cysteine/cystine from the microenvironment for glutathione synthesis needed to fight reactive oxygen species, due to their inability of diverting methionine‐derived metabolites toward cysteine synthesis. We show that via nutritional intervention alone, we can extend the survival of a mouse model of glioma, suggesting a time window for this type of intervention clinically.

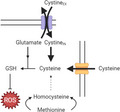

**Abbreviations**
BSO: buthionine sulfoximine
CBS: cystathionine β‐synthase
CTH: cystathionine gamma‐lyase
Cys: cysteine
Cyss: cystine
FDR: false discovery rate
GCLC: glutamate–cysteine ligase catalytic subunits
GCLM: glutamate–cysteine ligase modifier subunit
Gln: glutamine
GSH: glutathione
HRP: horseradish peroxidase
IDH1: isocitrate dehydrogenase 1
LC/MS: liquid chromatography/mass spectrometry
PAG: propargylglycine
ROS: reactive oxygen species
SAH: *S*‐adenosylhomocysteine
SAM: *S*‐adenosylmethionine
SCID: severe combined immunodeficient
TCA: tricarboxylic acid cycle
TS: transsulfuration

## 1 Introduction

The metabolism of tumor cells depends on many factors, notably, nutrient availability from the microenvironment and oncogenic mutations. Others have studied whether restricting the nutrient availability from the microenvironment can reduce the distinctive high proliferation rate of Myc‐driven tumors, which require glutamine [1, 2, 3], or melanoma cells, which require leucine [4] or serine [5]. Although such dietary interventions have been explored recently, with the goal to ‘starve’ cancer cells [6, 7], their nutrient requirements and the mechanisms underlying their dependence on specific metabolites obtained from the environment are not fully understood.

Gliomas are brain tumors that can have an aggressive phenotype and currently have no curative treatment. The potential of incorporating a dietary plan into the treatment of this disease is highly desirable for patients. In clinical studies of gliomas, one of the most popular diet as adjuvant therapy is the ketogenic diet [8], with over 12 clinical trials currently exploring that type of intervention (NCT03451799, NCT03328858, NCT03160599, NCT02694094). However, no other interventional diets are being explored for this disease, in part due to a lack of preclinical evidence.

Gliomas harboring isocitrate dehydrogenase 1 (IDH1) mutations [9] have been reported to have a decreased ability to compensate for reactive oxygen species (ROS) [10], due to reduced IDH1‐wild‐type activity and concomitant low NADPH [11, 12]. Recent reports noted that upregulation of antioxidant pathways compensates for the increased ROS levels found in IDH1‐mutant tumors [13]. By tilting the balance toward increased oxidative stress [14], potentially via interventional diets, these findings provide a new framework to investigate alternative therapeutic strategies for gliomas.

Diets that restrict specific amino acids, particularly essential amino acids, have been explored in other cancers [6]. However, cysteine is a nonessential amino acid, as it can be synthetized from methionine through the transsulfuration (TS) pathway. The most abundant form of cysteine is cystine, which has plasma levels 10 times higher than cysteine [15]. Once cystine enters the cell it is reduced to cysteine, which then can be utilized for glutathione (GSH) synthesis, a major ROS scavenger. Alternatively, methionine can be converted into homocysteine through its sequential transformation into the metabolic intermediates *S*‐adenosylmethionine (SAM) and *S*‐adenosylhomocysteine (SAH). Subsequently, homocysteine can either be diverted into cysteine synthesis through the TS pathway or be remethylated to yield methionine and generate tetrahydrofolate. Hepatic tissue synthetizes almost half of the total pool of GSH from methionine‐derived cysteine [16], although the importance of the TS pathway is not limited to the liver. Indeed, experiments conducted in mice revealed 29% less GSH in brain presenting homozygous cystathionine β‐synthase (CBS) gene disruption [17]. However, it is not clear when this pathway plays a main role in supplying cysteine instead of being taken up from the environment [18].

Herein, we discover the inability of TS pathway to compensate for the lack of cysteine/cystine from the microenvironment, thus creating a metabolic bottleneck for glioma cells that affects their viability and growth. We demonstrate that glioma cells need cysteine for GSH biosynthesis to fight ROS and make proteins for growth. We then propose a new method to hypersensitize glioma cells to oxidative stress without using any drug treatment. Using a *in vivo* model of IDH1‐mutant glioma, we test the hypothesis that dietary cysteine/cystine restriction for cancer cells contributes to improved survival.

While the disruption of cysteine concentration is achieved transitory at the systemic level, and it is compensated over time, we propose that this time window could be exploited further to improve the survival of these mice with additional drugs. This study highlights how we can use dietary interventions to create metabolic vulnerabilities that can be exploited to design more efficient therapies.

## 2 Materials and methods

### 2.1 Cell culture

Cell lines utilized in this investigation: TS603 [19] was a gift of T. A. Chan (Memorial Sloan‐Kettering Cancer Center, USA); NCH1681 [20] was provided by C. Herold‐Mende (University Hospital Heidelberg, Germany); BT142 was purchased from ATCC^®^ (ACS‐1018™, Manassas, VA, USA). Cell lines were authenticated using DNA sequencing, the EPIC 450 Illumina platform to analyze the glioma methylome and the 1p/19q co‐deletion, western blots for IDH1‐mutant enzyme, and D‐2HG quantification via liquid chromatography/mass spectrometry (LC/MS) (Fig. S1). These cell lines have been grown only as 3D spheres and have been used and validated in other studies as well [21, 22, 23, 24].

All cell lines were grown in DMEM:F12 medium supplemented with antibiotics (penicillin–streptomycin), 1% N2 growth supplement, heparin sulfate (2 µg·mL^−1^), EGF (20 ng·mL^−1^), and FGF (20 ng·mL^−1^). For experiments involving seeding the cells in medium containing ^13^C tracers or lacking cysteine/cystine, neurospheres grown in DMEM:F12 (including antibiotics, N2, heparin sulfate, EGF, and FGF) medium were collected, centrifuged, and resuspended in PBS. Cells were then spun down, the supernatant was discarded, the pellet was resuspended again in PBS, and neurospheres were mechanically disaggregated for cell counting and subsequently seeded in the corresponding medium containing ^13^C metabolites.

Medium lacking cysteine and cystine was provided by Thermo Fisher (Waltham, MA, USA) upon request. [C3‐^13^C]‐cysteine (Cambridge Isotopes Laboratories, Tewksbury, MA, USA; CLM‐1868) and [C3,3′]‐^13^C‐cystine (Cambridge Isotopes Laboratories; CLM‐520) were utilized for ^13^C tracing. Experiments involving *methyl*‐^13^C‐methionine (Cambridge Isotopes Laboratories; CLM‐206‐1), [C3‐^13^C]‐serine (Cambridge Isotopes Laboratories; CLM‐1572), and [U‐^13^C]‐glutamine (Cambridge Isotopes Laboratories; CLM‐1822) were performed in DMEM:F12 using in‐house made medium that lacked the metabolites utilized as ^13^C tracers.

### 2.2 In‐house made medium

We created 20×–100 000× solutions (according to solubility) of all the components described in the DMEM:F12 formulation. For compounds that required a non‐neutral pH to be soluble in water, a 1 N solution of NaOH or HCl was used to titrate the solutions. Components of the medium were added to reach the final concentrations described in the commercial version of DMEM:F12, but linoleic acid was included directly from the vial provided by the vendor. Distilled water was added to obtain the final volume desired. This medium was subsequently filtered, and the supplements described above were added, and it was filtered again. All the ^13^C probes were utilized in the same concentrations as those specified in the DMEM:F12 commercial formulation.

### 2.3 Sequencing IDH1 in genomic DNA

The sequence of IDH1 gene in genomic DNA of TS603 and NCH1681 cells was conducted as previously described [13]. In brief, the genomic DNA of cells was extracted using DNeasy Blood & Tissue Kit (QIAGEN, Germantown, MD, USA). The exon 4 of IDH1 gene was amplified using NEBNext High‐Fidelity 2× PCR Master Mix. The primers used in PCR were as follows: IDH1.F: 5′‐TGA GCT CTA TAT GCC ATC ACT GC‐3′; IDH1.R: 5′‐CAA TTT CAT ACC TTG CTT AAT GGG‐3′. The DNA sequence was analyzed by Sanger’s sequencing.

### 2.4 Methylation

Genome‐scale DNA methylation was analyzed using the Illumina Human Infinium Methylation EPIC BeadChip according to manufacturer’s instructions in the Cancer Genomics Research Laboratory (National Cancer Institutes, NIH), which quantifies more than 850K CpG‐sites at the single nucleotide resolution. The idat files were uploaded into the Heidelberg website for classification of cell lines using methylation profile [25].

### 2.5 Treatments and proliferation assays

A total of 50 000–100 000 cells/well were seeded in untreated 48‐well plates with 0.75 mL of culture medium in triplicate (all experiments were repeated at least twice). After treatment or cystine/cysteine deprivation, gliomaspheres were mechanically disaggregated, transferred to a vial and cell number and viability was assessed using the Vi‐CELL XR cell counter (Beckman Coulter, Brea, CA, USA). Glutathione and its adduct were added at a final concentration of 0.5 mm. *S*‐adenosylmethionine, homocysteine, cystathionine, and taurine were dissolved in PBS, filtered, and added to a final concentration of 0.1 mm. Nucleosides (100×; Millipore Sigma ES‐008‐D, St. Louis, MO, USA) were added directly to the cultured cells after filtration to a 1× final concentration. AGI5198 was dissolved in DMSO and used in a 10 µm final concentration. Buthionine sulfoximine (BSO) and trolox were dissolved in PBS and DMSO, respectively, at 0.25 mm final concentrations. Ferrostatin 1 was utilized at a final concentration of 2 µm. H_2_O_2_ and tert‐butyl hydroperoxide (tBH) were dissolved in PBS, filtered, and added to the wells to reach the final concentrations specified in the graphs.

### 2.6 DCFDA assay

Experiments were performed employing the DCFDA/H_2_DCFDA‐Cellular ROS Assay Kit (Abcam, Waltham, MA, USA; ab113851) according to the vendor’s protocol and analyzed with a Sony SA3800 spectral analyzer (Sony Biotechnology, San Jose, CA, USA). Flow cytometry analyses were performed at the NCI LGI Flow Cytometry Core supported by funds from the Center for Cancer Research. Data were processed and plots were generated with flowjo 10.6 (BD Biosciences, Ashland, OR, USA).

### 2.7 Apoptosis assay

Experiments were performed using the 7AAD Annexin V Apoptosis Detection Kit (Abcam; ab214663) according to the vendor’s protocol and analyzed with a FACSCalibur (Becton Dickinson, Franklin Lakes, NJ, USA). Data were processed and plots were generated with flowjo 10.6 (BD Biosciences).

### 2.8 Sample preparations for metabolomics

Cells were collected by centrifugation and washed twice with PBS, and the resulting pellet was stored at −80 °C until extraction. For extraction, cell pellets were thawed on ice and lysed by three cycles of freeze–thawing, including a 5‐min sonication process in an ice‐water bath during the thawing step. Then, 40 μL of the homogenate was put aside for protein quantification by the Bradford method for further normalization of the metabolite levels. Next, metabolites were extracted by mixing the lysate with methanol : chloroform at a final ratio of 1 : 2 : 2 (water : methanol : chloroform), thoroughly vortexed, and incubated in ice with agitation for 10 min. Then, samples were centrifuged at 13 750 ***g*** for 20 min at 4 °C. The two resulting phases (upper aqueous polar and lower organic lipid) were separated, and the protein interface was discarded. Polar extracts were dried under a stream of N_2_ and stored at −80 °C until metabolomics analysis was performed. Tumor tissue was first weighed while frozen for normalization purposes and subsequently homogenized using a bullet blender homogenizer in the same solvent mixture described above and further processed in the same way as cell extracts. Cell culture medium was collected after centrifugation of cells (300 rcf, 5 min), extracted in ice‐cold methanol, dried under N_2_, and resuspended in 180 μL of phosphate buffer (pH 7; 100 mm) in D_2_O (containing d‐TSP) and 1% NaN_3_ for NMR spectroscopic analysis.

### 2.9 NMR spectral acquisition and processing

All spectra were acquired at 25 °C on a Bruker Avance III 600 MHz spectrometer (Structural Biophysics Laboratory, NCI, Frederick, MD, USA) equipped with a cryogenically cooled probe. Single‐pulse ^1^H NMR experiments were performed using the noesygppr1d (topspin 3.5; Bruker Biospin, Billerica, MA, USA) pulse sequence for water suppression. For each spectrum, 128 scans were acquired, with a relaxation delay of 3 s, a spectral width of 10 800 Hz, and a time domain of 32K points. Spectra were referenced to the TSP internal standard signal (s, δ = 0.00 p.p.m.), zero‐filled to 64K points, phased, and baseline‐corrected using ACD Labs Spectrus Processor 2016, and an exponential line‐broadening function of 0.30 Hz was applied. For quantification, ^1^H NMR resonance signals were normalized to the TSP singlet located at 0.00 p.p.m. and corrected to either the total protein content as obtained from the Bradford assay, cell number, or tissue weight.

### 2.10 LC/MS acquisition and processing

LC/MS analysis was conducted with an Agilent 6545 MS combined with a 1290 Infinity II UHPLC system (Agilent Technologies, Wilmington, DE, USA). Only LC/MS‐grade solvents and additives purchased from Covachem, LLC (Loves Park, IL, USA), were used to prepare mobile phases and wash solutions. Wash cycles consisting of a strong wash (50% methanol, 25% isopropanol, and 25% water), weak wash (90% acetonitrile and 10% water), and seal wash (10% isopropanol and 90% water) were implemented to eliminate carryover between injections. Analytes were injected (8 µL) and resolved using an Infinity 1290 in‐line filter combined with an AdvanceBio Glycan Map 2.1 × 100 mm, 2.7 µm column (Agilent Technologies) set at 35 °C. The solvent buffers, consisting of mobile phase A (10 mm ammonium acetate in 88% water and 12% acetonitrile) and mobile phase B (10 mm ammonium acetate in 90% acetonitrile), were initially titrated with formic acid and ammonium hydroxide to pH 6.85. The linear gradient was executed at a flow rate of 0.3 mL·min^−1^, as follows: 100% B, 0.5 min; 95% B, 2.0 min; 60% B, 3.0 min; 35% B, 5 min; hold 0.25 min; 0% B, 6 min; hold 0.5 min; 100% B, 7.8 min. The mass analyzer acquisition conditions were as follows: drying gas temperature 250 °C, sheath gas temperature 325 °C, nebulizer 45 psig, skimmer 50 V, and octopole radio frequency 750 V. Mass spectra were acquired at 3.0 spectra·s^−1^ in negative electrospray ionization mode for a mass range from 72 to 1200 *m/z* using a voltage gradient of capillary 3000 V, nozzle 2000 V, and fragmentor 80 V.

Prior to preprocessing the datasets, pooled quality control samples were inspected for consistency of retention‐time (RT) shifts and signal degradation. Following acquisition, *m/z* spectra binning was performed by partitioning the *m/z* vs. RT matrices into fixed width using an Agilent masshunter profinder B.08.00. Bins were manually inspected to confirm consistent, reproducible integration for all analytes of interest across all samples. Target extraction of precursor *m/z* was performed using an in‐house compound library. Ion selection and alignment parameters were restricted to proton loss (H‐) only, 5.0 mDa mass range, and RT ± 0.4 min. Following preprocessing, the ion areas were reported for each sample and corrected to the area of sample‐specific internal standard, *p*‐nitrobenzoate (added at 90 pmol per sample during preparation).

The same acquisition procedure was followed for the ^13^C isotopically labeled samples. After alignment and identification of analytes of interest retention times a PCDL card was constructed using pcdl manager B.07.00 (Agilent). The chromatograms were introduced into the Agilent masshunter profinder B.08.00, and the PCDL card was used in the Batch Isotopologue Extraction routine with the following parameters: 99% ^13^C labeling, 20% peak height ion abundance criteria, mass tolerance of ±15 p.p.m. + 2 mDa with a threshold of 250 counts for the anchor and 1000 counts for the sum of the ion heights with a minimum correlation coefficient greater than 0.5. The corrected and raw intensity and percentages of isotopologues of the analytes of interest were obtained. Metabolite levels obtained from the LC/MS analysis described above are displayed either as the percentage of the specific isotopologues over the total pool of the metabolite for ^13^C‐tracing experiments or as protein/tissue‐normalized intensities.

### 2.11 D‐2HG quantification

#### 2.11.1 Sample preparation

Cells were collected by centrifugation and washed twice with PBS, and the resulting pellet was stored at −80 °C until extraction. For extraction, cell pellets were thawed on ice and lysed by three cycles of freeze–thawing, including a 5‐min sonication process in an ice‐water bath during the thawing step. Prior to extraction, 430 µL lysate was administered 100 µL of 70% MeOH (aq) internal standard solution—composed of 9.8 nmol·mL^−1^ U‐13C_5_‐D‐2‐hydroxyglutarate. Each sample was vortexed on BenchMixer (Benchmark Scientific, Edison, NJ, USA) at mid‐speed for 15 s and incubated in ice for 20 min on rotating mixer at mid‐speed. Upon extraction, 600 µL chilled LC/MS‐grade methanol was added to each sample lysate. Samples were vortexed with for 30 s and placed on Orbi‐blotter mixing rotator (Benchmark Scientific) at max speed to incubate in ice bath for 10 min. Samples were vortexed and administered 300 µL chilled (−20 °C) acetonitrile (ACN) each and returned to ice bath on rotator for 30 min to precipitate protein. All samples remained in ice bath until final centrifugation and collection. Samples were centrifuged at 14 200 ***g*** for 20 min at 4 °C. Following centrifugation, 90% of the supernatant volume was transferred to new tube while aspirating slowly to not disrupt protein pellet. Extracts were placed under N_2_ until completely dry and then stored at −80 °C. Dried samples were reconstituted in 100 µL of 100% H_2_O (LC/MS grade), homogenized via vortexing (15 s on the high speed) and centrifuged at 10 000 ***g*** for 5 min at 4 °C. Samples were transferred to glass LC vial with deactivated 250‐µL inserts (Agilent Technologies).

#### 2.11.2 LC/MS analysis of D‐2HG

D‐2HG quantification was achieved with Hi‐Resolution Agilent 6545 Quadrupole Time‐of‐Flight (QTOF) Liquid Chromatography Mass Spectrometry with Liquid Chromatography (LC/MS) as previously described [23]. A Zorbax Eclipse RRHD 2.1 × 100 mm, 1.8 µm column (Agilent Technologies) at temperature of 40 °C. The mobile phase was composed of 2 mm ammonium acetate in 100% H_2_O (Fisher Optima, Hampton, NH, USA) pH 3.5 titrated with formic acid (LC/MS grade; Covachem) and B 95% MeOH (Covachem)/5% ACN (Fisher UHPLC Optima) and flow rate of 0.200 mL·min^−1^. The gradient was initially 3% B hold for 6 min; ramp to 5% B min over 1 min; ramp to 99% B over 5 min, hold for 1 min and equilibrate at initial conditions for 2.5 min. The MS conditions were as follows: 3.8 spectra·s^−1^, V Cap 2600 V, nozzle 1000 V, fragmentor 80 V, skimmer 37 V, drying cap gas flow at 8 L·min^−1^ 300 °C, sheath gas flow 10 L·min^−1^ at 250 °C, nebulizer 45 psig, and collision energy 0 V.

#### 2.11.3 Quantification

Standard curve was determined by plotting the ratio for peak area to internal standard. The calibration curve for D‐2HG standards ranged from 0 to104 nmol·mL^−1^. Graphs were generated in graphpad prism v7.05 (GraphPad Software, San Diego, CA, USA). D‐2HG concentrations were normalized to the sample protein content (mg) measured via Bradford protein quantification.

### 2.12 Metabolomic analyses

Volcano plots were generated from the datasets obtained from the LC/MS analysis of the polar extracts of the three cell lines. Samples were labeled as control or cysteine/cystine‐free. Then, *P* values obtained by a *t*‐test followed by Welch correction were adjusted for multiple comparisons by the false discovery rate (FDR) method. In addition, fold changes (FC) were computed for each metabolite to generate the plot, including −Log(FDR‐corrected *P* values) vs Log_2_(FC). Thresholds used to highlight dysregulated variables were Log_2_(FC) > 1 or < −1 and FDR‐corrected *P* values < 0.05. Volcano plots and PCA data were obtained by metaboanalyst 4.0 [26].

### 2.13 Western blots

Samples were harvested and centrifuged at 300 ***g*** for 3 min, washed with PBS, and lysed in a solution of the radioimmunoprecipitation (RIPA) buffer system (Santa Cruz Biotechnology, Dallas, TX, USA). After a 30‐min incubation period and a brief sonication on ice, lysates were centrifuged at 14 000 ***g*** for 10 min at 4 °C. Total protein concentration of supernatants was then determined by BCA assay (Thermo Scientific, Waltham, MA, USA). Samples were prepared for electrophoresis as 20–50 µg of protein, Laemmli sample buffer, and dithiothreitol. Protein samples were boiled at 95 °C for 5 min and loaded into either 8–16% or 10% Mini‐Protean TGX Precast Gels for separation by electrophoresis. Then, proteins were transferred to nitrocellulose membranes using the Trans‐Blot Turbo system. Blots were blocked for 1 h at room temperature with 5% fat‐free milk or milk‐free blocking buffer (for analysis of phosphorylated proteins) and incubated overnight at 4 °C with primary antibodies. Primary antibodies included p‐GCN2 (Abcam; ab75836), p‐eIF2α (Cell Signaling, Danvers, MA, USA; #9721), α‐tubulin (Cell Signaling; #2144), β‐actin (Abcam; ab8227), CBS (Abcam; ab135626), cystathioninase (Cell Signaling; #30068), glutamate–cysteine ligase regulatory subunit (Proteintech, Rosemont, IL, USA; 14241‐1‐AP), IDH1 R132H (Millipore; #MABC171), and MTHFD1 (Proteintech; 10794‐1‐AP). Subsequently, the membrane was washed with Tris‐buffered saline + Tween‐20, incubated with horseradish peroxidase (HRP)‐conjugated secondary antibodies for 1 h at room temperature and treated with Clarity Max Western ECL substrate. Blots were imaged on the Chemidoc MP imaging system (Bio‐Rad, Hercules, CA, USA).

### 2.14 GSH quantification

GSH levels were computed using the GSH‐Glo™ Glutathione Assay (Promega, Madison, WI, USA) according to the protocol provided by the vendor. Then, 24‐well plates were seeded at 200 000–300 000 cells in 2 mL for 4 days. To normalize GSH levels, 0.5 mL was taken from each well prior to performing the assay, and the cells were counted by a ViCell XR automatic cell counter (Beckman Coulter, Indianapolis, IN, USA).

### 2.15 Animal studies

The intracranial orthotopic mouse model harboring the IDH1‐mutant glioma cell line NCH1681 was established according to approved animal study proposal NOB‐008 by the National Cancer Institute–Animal Use and Care Committee. Briefly, cells were harvested, washed with PBS, and counted. The resulting pellet was resuspended in Hank’s balanced salt solution, and 2.5 μL of the cell suspension (500 000 cells/mouse) were injected stereotactically into the striatum of 6–8‐week‐old female severe combined immunodeficient (SCID) mice (Charles River Frederick Research Model Facility) using a stereotactic device. Before the diet was initiated, mice were randomized and split into the control and the diet groups.

The number of animals per group was determined by using G*Power 2. The sample size (number of animals) was computed via *a priori* methods of calculation, assuming an alpha error probability of 0.05, power level of 0.95, and difference in mean survival between groups of 9 days. Neurological symptoms of mice were monitored daily to assess tumor growth; specifically, an external‐independent researcher assessed the health of the mice twice a day without previous knowledge of the experiment (blinded to the treatment). Once this researcher determined that a mouse was reaching end point, in view of the symptomatology, that mouse was euthanized. Symptoms include animal experiencing rapid weight loss (> 15%, monitored daily), debilitating diarrhea, rough hair coat, hunched posture, labored breathing, lethargy, persistent recumbence, significantly abnormal neurological signs, bleeding from any orifice, self‐induced trauma, impaired mobility, moribund, or otherwise unable to obtain food or water.

To compare survival curves, the log‐rank (Mantel–Cox) test was used. Diets supplied to the mice were A05080217 (l‐amino acid rodent diet without added cystine) and A18110101 (l‐amino acid diet with 2 g l‐cysteine and 2 g l‐cystine per kg) from Research Diets Inc. (New Brunswick, NJ, USA). Prior to initiation of the experiment, control mice were fed with both diets to check whether there was active ingestion of food. Accordingly, food and animal weight were monitored twice a week, and stool was examined visually for incorporation of food dyes. Tissue was submitted to Histoserv, Inc. (Germantown, MD, USA) for analysis.

### 2.16 Animal housing and handling

Eighteen 6–8‐week‐old female SCID mice were obtained from Charles River Frederick Research Model Facility. Mice were housed in sterile cages of five compatible cage mates per cage, in a controlled environment of constant temperature (21 °C) and humidity (50%) with 12 : 12 light/dark schedules. The hygienic status was specific pathogen‐free according to National Cancer Institute–Animal Use and Care Committee. Animals were cared for every day, and food and water were provided *ad libitum*. All animals were marked with a specific number by ear tags on arrival. All animal experiments were approved by the National Cancer Institute Animal Care and Use Committee (NCI ACUC) and performed in accordance with the National Institute of Health Guide for the Care and Use of Laboratory Animals (NIH Publications No. 80‐23).

### 2.17 ^13^C tracing *in vivo*

^13^C‐tracing experiments *in vivo* were performed as previously described [27, 28] on the same mice utilized for survival analysis. [^13^C‐U]‐glutamine was prepared as a 36.2 mg·mL^−1^ stock solution in sterile PBS and injected (200 μL, 7.24 mg) into the tail vein at 15‐min intervals for three times (total = 142 μmol) just prior to mice reaching end point. Mice were euthanized 15 min after the last injection (45 min from the first injection). Tumors were separated from the brain, then both tumor and normal tissues were gently blotted by rapidly tapping the tissue onto a cloth and were immediately flash‐frozen in liquid nitrogen.

### 2.18 Plasma analysis

Blood was collected from the tail vein of the mice in lithium–heparin collection tubes (Sarstedt, Germany; #41.1393.105). Approximately 35 µL of blood was centrifuged, according to the tube manufacturer’s instructions, at 4 °C, and the clear plasma fraction was transferred to a clean microtube. Subsequently, plasma was extracted in a water : methanol : chloroform mixture, centrifuged for 20 min at 4 °C and 23 240 ***g***, and the resulting upper hydrophilic phase was then transferred to a clean vial and dried under a stream of N_2_. Dried sediments were resuspended in methanol and injected into the LC/MS system for global profiling.

### 2.19 Immunohistochemistry

When mice reached end point, tissue was collected and stored in paraformaldehyde at 4 °C. Tissue was submitted to Histoserv, Inc. for analysis together with the antibodies. Slides were deparaffinized and hydrated through graded alcohols to distilled water, followed by antigen retrieval. They were then blocked with hydrogen peroxide and a blocking serum. Next, the slides were incubated with the primary antibody, a secondary antibody, and HRP‐conjugated streptavidin. Finally, the slides were developed using 3,3′‐diaminobenzidine and counterstained with hematoxylin. All the incubations were carried out at room temperature, with Tris‐buffered saline + Tween‐20 used as a washing buffer. Immunostaining was quantified using imagej software (National Institutes of Health, Bethesda, MD, USA).

### 2.20 Statistical analysis and graphs

Statistical significance was computed with R Core Team (Free Software Foundation, Inc., Boston, MA, USA) or graphpad prism v7.05 (GraphPad Software). Multivariate analysis was performed using metaboanalyst 4.0 [29] and in‐house R scripts. For each figure, ‘*n*’ refers to the number of biological replicates.

## 3 Results

### 3.1 Cysteine and cystine deprivation halt the growth and reduce the viability of gliomas by downregulating protein translation and glutathione synthesis

**Fig. 1:**
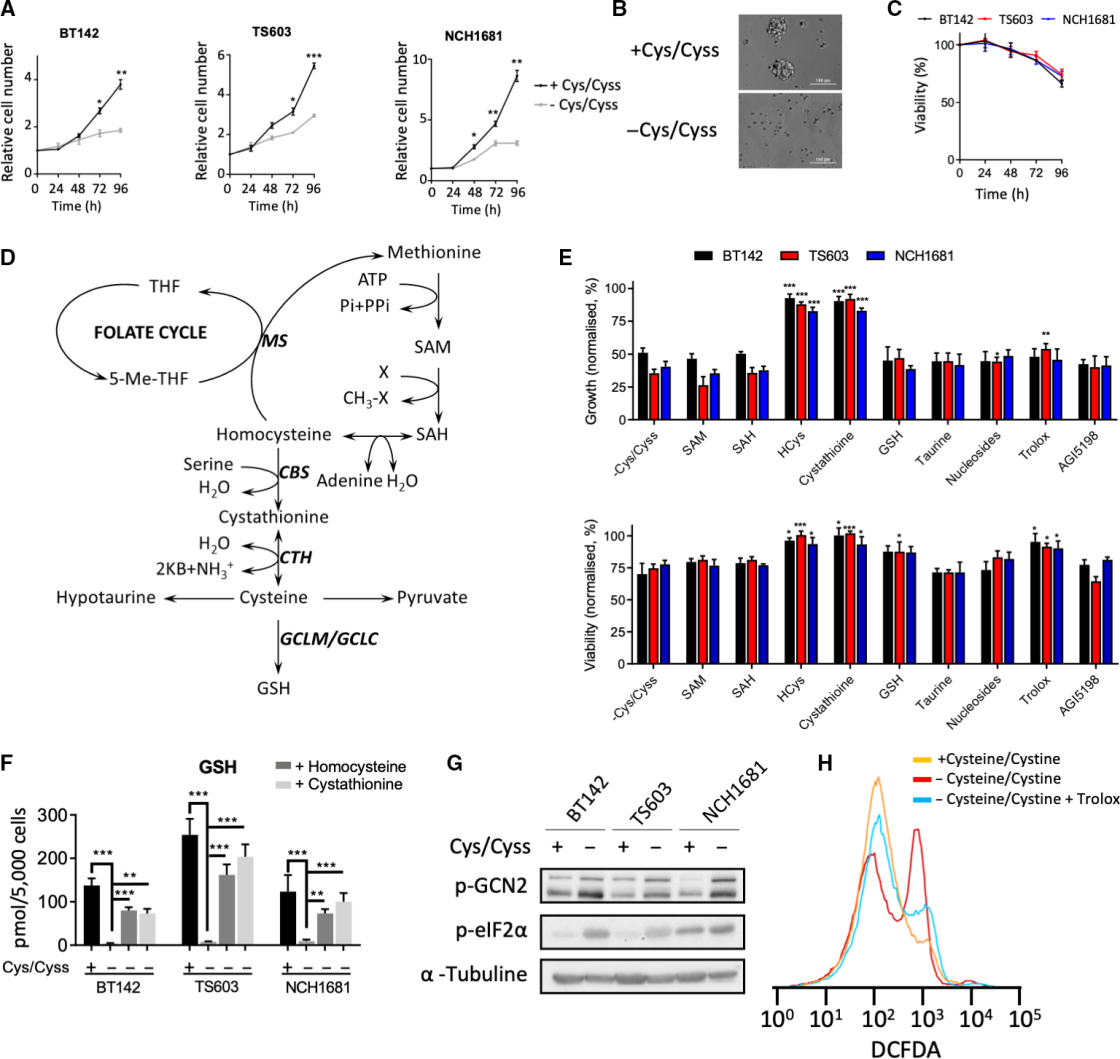
Cysteine and cystine deprivation halt growth and reduce viability in glioma through reduced protein translation and glutathione synthesis. (A) Growth rate of IDH1‐mutant glioma cell lines under cysteine/cystine (Cys/Cyss) deprivation conditions over 96 h. Data are shown as the ratio of viable cells to cells seeded (2‐way ANOVA followed by Sidak’s multiple comparison test, *P < 0.05; **P < 0.005; ***P < 0.001; *n* = 4, data displayed as mean ± SD for each time point and condition). (B) Neurosphere formation after 96 h of incubation in the same medium. BT142 cells displayed as a representative picture (experiment was done in triplicate for each cell line); scale bar is 100 μm. (C) Viability after 96 h in medium lacking cysteine/cystine as the percentage over the viability of cells in control conditions (*n* = 3, data displayed as mean ± SD for each time point and condition). (D) Metabolic pathways related to cysteine (THF, tetrahydrofolate; MS, methionine synthase; CBS, cystathionine beta‐synthase; CTH, cystathionine gamma‐lyase; GCLM, glutamate–cysteine ligase modulatory subunits; GCLC, glutamate–cysteine ligase catalytic subunits). (E) Rescue experiments involving the addition of different metabolites and agents to cells grown without cysteine/cystine. Data displayed as control‐normalized growth (top panel) and viability (bottom panel) as the mean ± SD for *n* = 3; *P < 0.05; **P < 0.005; ***P < 0.001; each condition was compared with the −Cys/Cyss values by using a two‐tailed Student’s *t*‐test. (F) Glutathione (GSH) levels in complete medium (+) and medium lacking cysteine/cystine (−) plus 0.1 mm homocysteine and cystathionine (2‐way ANOVA followed by Dunnett’s multiple comparison test for −cysteine/cystine vs all groups, **P < 0.005; ***P < 0.001 was used and data are displayed as the mean ± SD for *n* = 3). (G) Representative western blot of p‐GCN2 and p‐eIF2α in both complete and cysteine/cystine‐free media for the three cell lines investigated (quantitative data for *n* = 3 is displayed in Fig. S2C,D). (H) Intracellular ROS levels assessed by the DCFDA assay in glioma cell lines under cysteine/cystine deprivation and treated with 0.25 mm trolox for 96 h. BT142 cell line data are shown as a representative diagram and data from other cell lines are shown in the Fig. S1E.

Three glioma cell lines, that is, BT142 (IDH1^mut^ high‐grade astrocytoma), TS603 (IDH1^mut^ oligodendroglioma and 1p/19q codeleted), and NCH1681 (IDH1^mut^ high‐grade astrocytoma) were cultured in DMEM:F12, which either contained or lacked both cysteine and cystine. The absence of those amino acids had an antiproliferative effect (Fig. 1A), which was more intense in the BT142 cell line, limiting its growth to 50% over the course of 96 h. Moreover, cells were unable to form neurospheres (Fig. 1B and Fig. S2A), and their viability was reduced by approximately 30% in 96 h (Fig. 1C). At 72 h, all cell lines displayed a significant decrease (*P* < 0.001 for all cell lines) in viability, and TS603 even had a significant decrease in viability at 48 h (*P* = 0.038). To examine the key factors involved in both the antiproliferative effect and the reduction in viability resulting from cysteine/cystine deprivation, we conducted rescue experiments by treating the cells with downstream metabolites of cysteine or methionine (Fig. 1D,E). We also employed an inhibitor of the IDH1‐mutant enzyme (AGI5198) because these cell lines harbor this mutation. This agent did not alter the deleterious effect of cysteine/cystine deprivation (Fig. 1E). These experiments revealed that only homocysteine and cystathionine could restore the cell growth; thus, we explored the link between their supplementation and an increase in cysteine availability by checking the levels of GSH (Fig. 1F) and the expression of glutamate–cysteine ligase modifier subunit (GCLM) (Fig. S2B), which acts as a sensor of cysteine levels [30] in GSH synthesis. Both GSH and the expression of GCLM were reduced under cysteine/cystine deprivation, and their levels recovered after adding homocysteine and cystathionine (Fig. 1F), revealing that both metabolites are cysteine donors. Amino acid deprivation has been shown to inhibit protein synthesis in cancer [31], and because cysteine is also a proteinogenic amino acid, we evaluated the capacity of protein synthesis in cells subjected to cysteine/cystine‐deprived conditions. We examined the levels of phosphorylated eIF2α (p‐eIF2α) and GCN2 (p‐GCN2), two proteins involved in the stress response to amino acid starvation, and uncharged t‐RNAs [32, 33]. p‐eIF2α and p‐CGN2 levels were both significantly higher in the samples grown in the absence of cysteine/cystine (Fig. 1G, Fig. S2C,D), indicating that this nutrient deprivation reduces the protein synthesis activity. Moreover, both homocysteine and cystathionine recovered cell viability together with GSH and trolox, an antioxidant analogue of vitamin E, suggesting that the loss in viability was linked to the decreased ability of these cells to buffer the naturally occurring ROS (Fig. 1H and Fig. S2E) in the absence of cysteine/cystine from the microenvironment. While we were able to rescue the viability by exogenous addition of GSH in culture media, the ability of the GSH added to protect the cells against ROS was constrained by its poor cellular permeability [34]. Indeed, the intracellular levels of GSH along with cell viability could be further increased (Fig. S2F,G) by treatment with an esterified analogue of GSH that is more permeable [35].

Tumor cells usually display higher levels of oxidative stress [36], which can engage cancer cells in a ROS‐dependent death. We explored the mechanism of cell death by treating the cells with ferrostatin‐1 at 2 µm [37] at cell seeding time (Fig. S2H), an inhibitor of ferroptosis, and analyzing apoptotic markers (Fig. S2I,J). Cell viability did not recover after treatment with ferrostatin 1 (Fig. S2G), and an increase in apoptotic and preapoptotic cells was detected (Fig. S1I,J) after 96 h. Accordingly, the mechanism of cell death observed in these cell lines under cysteine/cystine depletion can be assigned to apoptosis, a process that can be triggered by high levels of ROS [38, 39]. Thus, the antiproliferative effects observed in our study occurred in part from cells’ inability to synthesize protein as a consequence of cysteine/cystine deprivation, whereas the loss of viability was attributed to the cells’ incapacity to fight ROS.

### 3.2 The transsulfuration pathway is not able to support GSH synthesis upon cysteine/cystine deprivation in gliomas

**Fig. 2:**
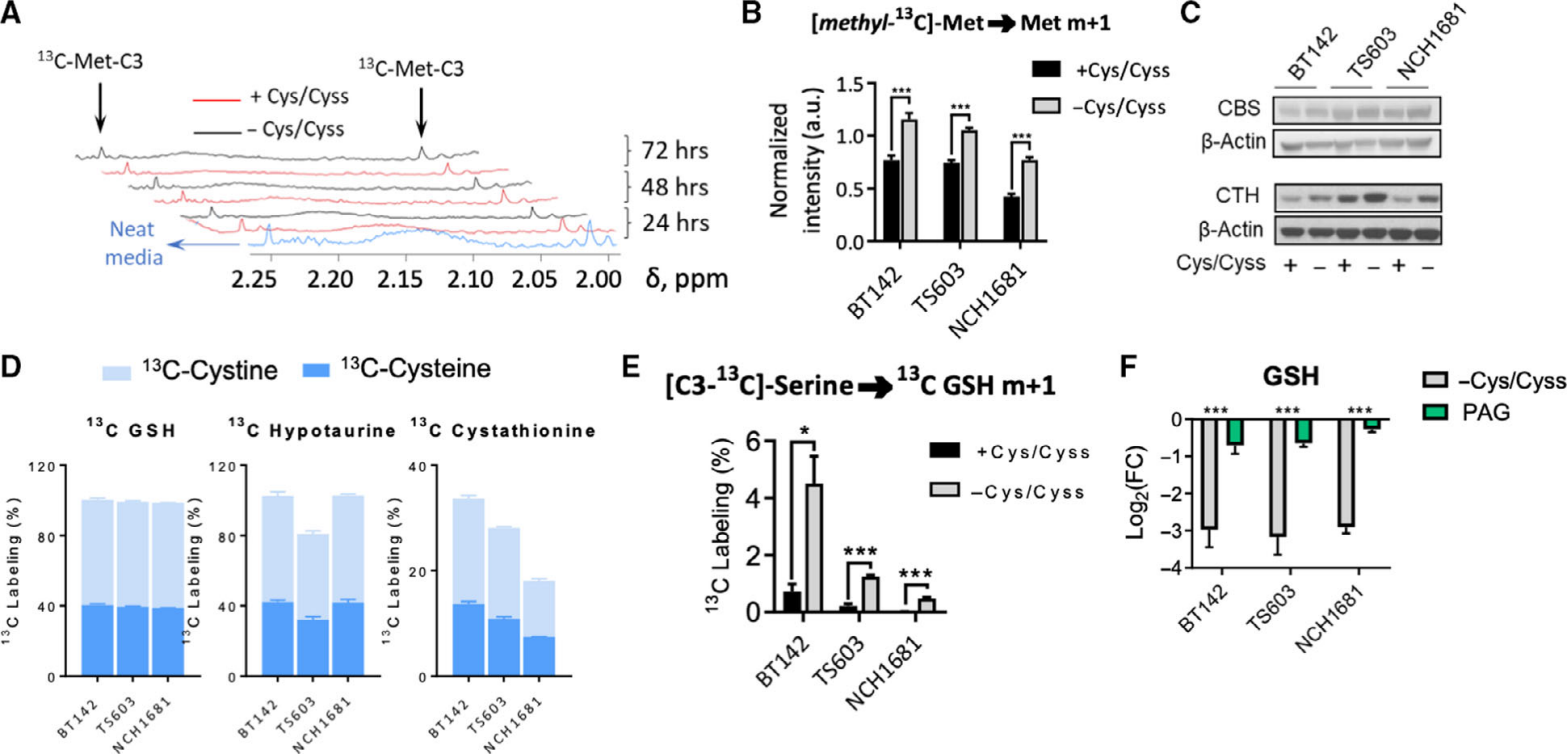
The TS pathway does not contribute to GSH synthesis in gliomas. (A) ^1^H NMR spectra of media over time, including assignments for *methyl*‐^13^C‐methionine. Representative spectra shown as normalized intensities to the trimethylsilylpropanoic acid (TSP) signal (δ = 0.00 p.p.m.) and cell number (experiment was done in triplicate for each cell line). (B) *Methyl*‐^13^C‐methionine levels in cells seeded in medium containing this tracer (data displayed as bar plots; mean ± SD, *n* = 3; ***P < 0.001, two‐tailed Student’s *t*‐test). (C) Representative western blot of CBS and CTH (quantitative data for *n* = 3 is displayed in Fig. S2A). (D) ^13^C‐tracing experiments displaying [C3‐^13^C]‐cysteine and [C3,3′‐^13^C]‐cystine incorporation into cystathionine, glutathione (GSH), and hypotaurine (*n* = 3 samples per cell line and ^13^C probe, data displayed as bar plots; mean ± SD). These experiments were conducted separately for each tracer (^13^C‐cysteine and ^13^C‐cystine). (E) Percentage of m+1 isotopologue over the total pool of GSH from [C3‐^13^C]‐serine (data displayed as bar plots; mean ± SD, *n* = 3; *P < 0.5; ***P < 0.001, two‐tailed Student’s *t*‐test). (F) Decrease of GSH levels (data displayed as bar plots of Log_2_(Fold Change, FC), mean ± SD, *n* = 5; ***P < 0.001, two‐tailed Student’s *t*‐test) for both PAG treatment and cysteine/cystine deprivation displayed.

Having demonstrated that exogenous cysteine is the limiting factor for GSH synthesis to combat ROS, we explored if the TS pathway may compensate this nutritional restriction. We first explored whether methionine availability limited the cells’ ability to upregulate the TS pathway and the concomitant cysteine synthesis. Glioma cells were seeded in media containing [*methyl*‐^13^C]‐methionine and the levels of this metabolite were investigated both in the media and within the cells in full and cysteine/cystine‐lacking medium. Through NMR analysis, we identified the ^13^C‐*methyl* resonance signal from methionine in the media even after 72 h, which indicated that methionine availability is not responsible for restricting cell growth even in the absence of cysteine/cystine (Fig. 2A). Concurrently, we detected the accumulation of *methyl*‐^13^C‐labeled methionine in cysteine/cystine‐deprived cells (Fig. 2B). Methionine is a proteinogenic amino acid, and the downregulation of protein synthesis (Fig. 1G), likely increases the intracellular pool of methionine [31]. We also evaluated the potential upregulation of enzymes connecting the methionine cycle with cysteine synthesis in order to compensate for the lack of cysteine/cystine. Expression of the first enzyme involved in the TS pathway, CBS was not significantly affected (Fig. 2C and Fig. S3A), which reveals the inability of the cells to upregulate the diversion of metabolic intermediates from the methionine cycle towards the TS pathway, even though the subsequent enzyme, cystathionine gamma‐lyase (CTH), was significantly upregulated (Fig. S3A).

Additionally, we investigated the contribution of cysteine and cystine to derived metabolites through [C3‐^13^C]‐cysteine and [C3,3′‐^13^C]‐cystine labeling, respectively, and LC/MS experiments. After 48 h, nearly 100% of GSH was derived from exogenous cysteine/cystine (Fig. 2D). We also observed an active reverse reaction of CTH, which was inferred from the labeling of cystathionine from ^13^C‐labeled cysteine and cystine, and the dependence of hypotaurine synthesis on exogenous cysteine/cystine. To probe the activity of the TS pathway, we incubated our cells in [C3‐^13^C]‐serine for 48 h, which revealed a labeling of < 1% in GSH in the presence of cysteine and cystine (Fig. 2E) and an upregulation in media lacking both amino acids. This increased labeling of GSH from serine may arise from the increased activity of CBS that can yield cysteine through the subsequent reaction of CTH, which is upregulated (Fig. 2C). Moreover, glioma cells treated with 1 mm propargylglycine (PAG) (Fig. 2F, Fig. S3B,C) for 48 h experienced a slight depletion in GSH levels, compared to the 10‐fold decrease (Fig. 2F) obtained after cysteine/cystine deprivation. These analyses revealed that the TS pathway is not intensively active in these cells, which rely almost completely on exogenous cysteine/cystine for GSH synthesis.

### 3.3 Global metabolic consequences of cysteine and cystine deprivation

**Fig. 3:**
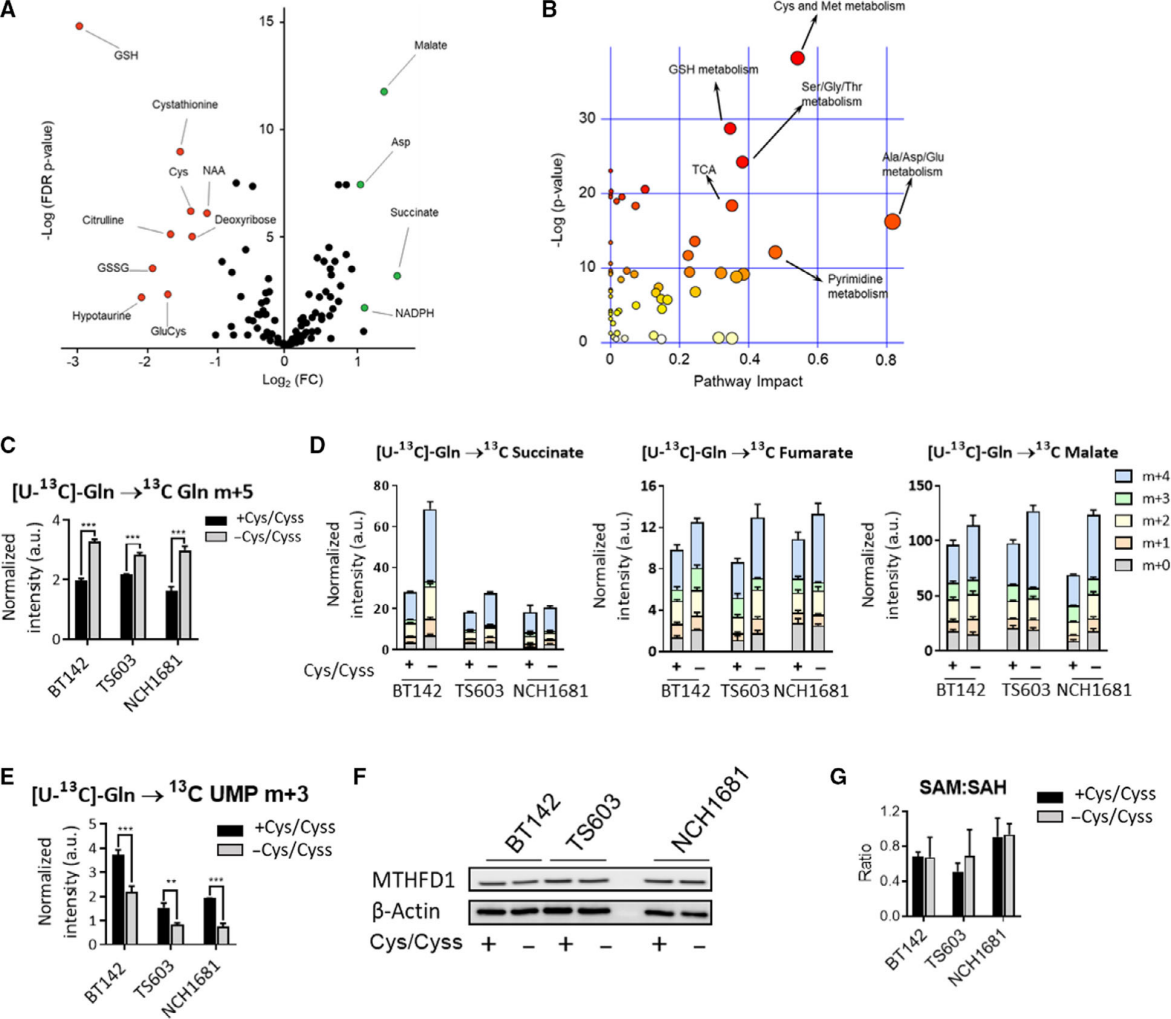
Global metabolic consequences of cysteine and cystine deprivation. (A) Volcano plot displaying the Log_2_(Fold Change, FC) vs Log(False Discovery Rate, FDR *P* value) for all the metabolites identified by LC/MS. Metabolites highlighted in green (upregulated upon cysteine/cystine deprivation) or red (downregulated) have Log_2_(FC) > 1 or < −1 and an FDR *P* value < 0.05 (5 replicates per cell line and per condition). (B) Pathway analysis performed on the metabolites levels computed depicts the major pathways affected. (C) ^13^C incorporation from [U‐^13^C]‐glutamine into cellular glutamine pools, and (D) tricarboxylic acid cycle (TCA) metabolites displayed as the contribution of each isotopologue to the total metabolite pool. ^13^C‐tracing experiments are displayed as bar plots; mean ± SD, *n* = 3 (***P < 0.001, two‐tailed Student’s *t*‐test). (E) ^13^C incorporation from [U‐^13^C]‐glutamine into uridine monophosphate (UMP) as the m+3 isotopologue (bar plots as mean ± SD, *n* = 3. *P < 0.05; **P < 0.005; ***P < 0.001, two‐sided Student’s *t*‐test). (F) Representative western blot of methyltetrahydrofolate dehydrogenase 1 (MTHFD1) as a marker of folate cycle activity. (G) Ratio of SAM and SAH for the 3 IDH1‐mutant glioma cell lines computed from the unlabeled global profiling experiment (*n* = 5, bar plots displaying mean values ± SD).

Next, we explored the metabolic changes resulting from this nutrient deprivation condition after 48 h (Fig. 3A), before cellular viability is affected (Fig. 1C); thus, we could analyze the metabolism without the potential confounding factors attributable to the mechanisms of cell death. The main metabolites affected were those directly related to the pathways that include cysteine/cystine, as well as those involved in related metabolic routes, such as the tricarboxylic acid (TCA) cycle (Fig. 3B). In order to investigate the metabolic consequences of cysteine/cystine deprivation on the TCA, we performed a ^13^C‐tracing experiment that involved seeding glioma cell lines in [U‐^13^C]‐glutamine to track its incorporation in downstream metabolites. We observed an accumulation of m+5 glutamine (Fig. 3C) along with an increased flux of glutamine toward the TCA cycle, as it is no longer needed for GSH synthesis (through glutamate conversion) or cystine import [40]. Accordingly, glutamate‐derived metabolites involved in the TCA cycle appear to be upregulated (Fig. 3D). In contrast, nucleotide synthesis from glutamine was downregulated (Fig. 3E), possibly due to halted cell proliferation, which decreased the demand for nucleotide biosynthesis.

The methyl group of methionine can also be transferred to nucleotides lipids and proteins [41, 42] and the resulting metabolite, SAH, can be further transformed into homocysteine or be remethylated to generate methionine. Accordingly, we used metabolomics analysis to determine whether the total levels of metabolites involved in these related pathways would be affected by cysteine/cystine deprivation. Global profiling experiments using LC/MS and analysis of MTHFD1 levels did not reveal any effect on the folate metabolism (Fig. 3F, Fig. S4A,B) neither on the synthesis of methyl donors that was assessed by the ratio of SAM to SAH (Fig. 3G) as an indicator of cellular methylation capacity [43].

In our metabolomic analysis, citrulline levels were also significantly lower after 48 h of cysteine/cystine deprivation, but we could not detect any additional metabolites from the urea cycle of the arginine biosynthesis pathway. However, arginine levels in NCH1681 were significant higher under cysteine/cystine deprivation (Fig. S4C).

### 3.4 Cysteine/cysteine‐free diet sensitizes glioma cells to oxidative stress *in vitro* and increases survival in a mouse model of glioma

**Fig. 4:**
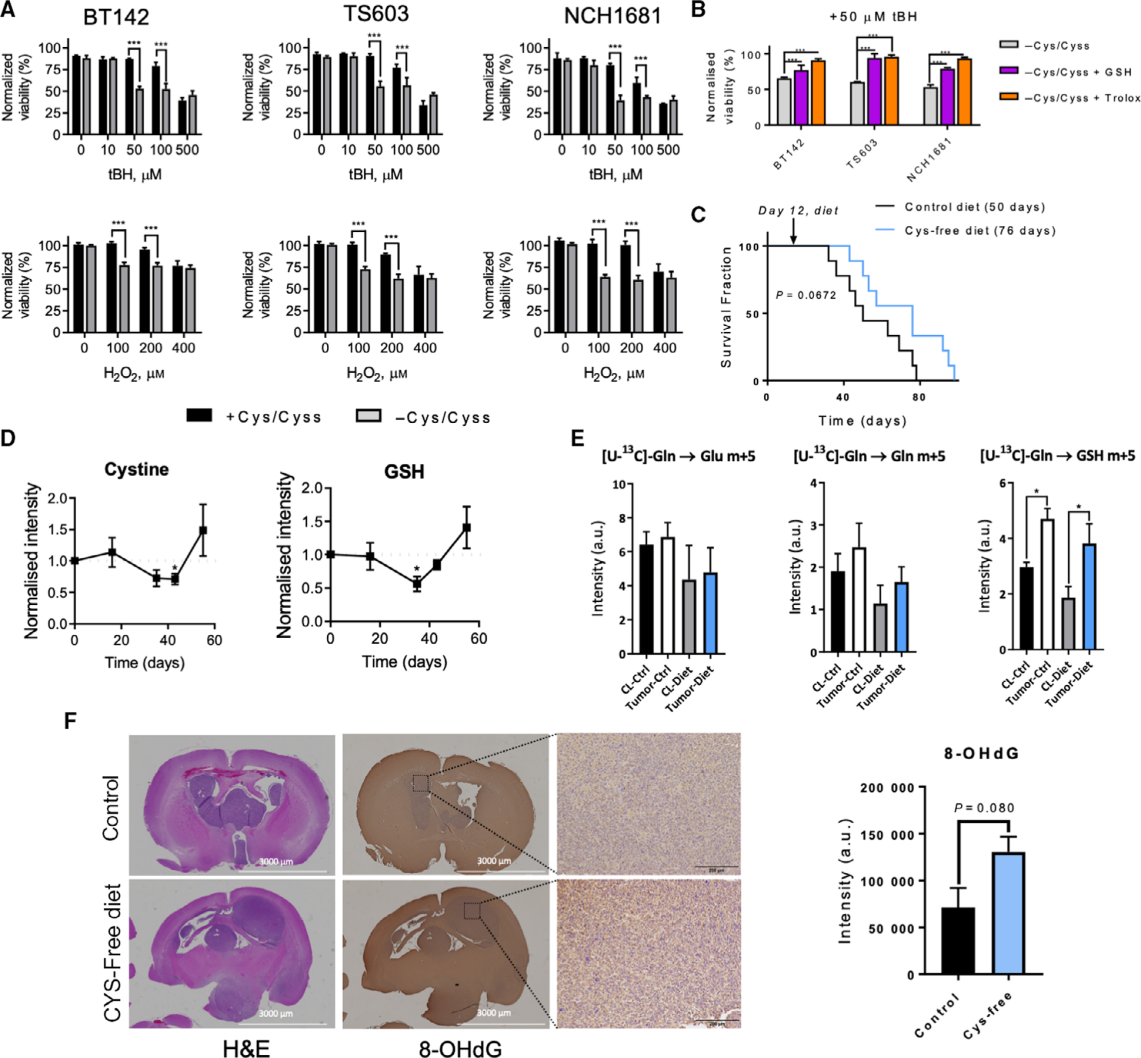
Cysteine/cysteine‐free diet sensitizes glioma cells to oxidative stress *in vitro* and increases survival in a mouse model of glioma. (A) Titration experiments involving tBH or H_2_O_2_ (data are mean ± SD, *n* = 3; ***P < 0.001; 2‐way ANOVA followed by Sidak’s multiple comparison test). (B) Normalized viability of the 3 IDH1 mutant glioma cell lines treated with 50 µm of tBH for 24 h in media spiked with 0.1 mm of glutathione (GSH) or 0.25 mm trolox (bar plots as mean ± SD, *n* = 3. *P < 0.05; **P < 0.005; ***P < 0.001, two‐sided Student’s *t*‐test). (C) Survival analysis of intracranial glioma mouse model under both diets (*n* = 9 mice per cohort, *P* value obtained from a Mantel–Cox test, and median survival displayed between brackets). (D) Plasma levels of GSH and cystine normalized to those computed for the control group (dotted line, reference values of the control group; metabolite levels for the cysteine/cystine‐free diet group are shown as mean ± SEM; *n* = 5; *P < 0.05 by two‐tailed Student’s *t*‐test with Welch’s correction). (E) Glutamate, glutamine, and GSH m+5 levels in tumor tissue and contralateral (CL) regions of mice fed with the control (Ctrl) and cysteine/cystine‐free diets resulting from bolus injection of [U‐^13^C]‐glutamine. Data are mean ± SEM of the normalized intensities to tissue weight (*n* = 3–5. *P < 0.05 by a one‐way ANOVA followed by Tukey’s multiple comparisons test). (F) Immunohistochemical analysis of hematoxylin and eosin (H&E)‐ and 8‐hydroxydeoxyguanosine (8‐OHdG)‐stained tissues, including quantification of 8‐OHdG (bars display mean ± SD, *n* = 3 mice, *P*‐value displayed from two‐tailed Student’s *t*‐test with Welch’s correction). Scale bar is 3000 μm for the left and middle panels and is 200 μm for the right panels.

To exploit the requirements of cysteine/cystine therapeutically, we tested whether our nutrient restriction sensitizes these cancer cell lines to ROS and induces oxidative stress, as this approach has been shown to effectively induce the death of other types of cancer cells [44, 45]. Thus, we conducted short titration experiments with tBH and H_2_O_2_ (Fig. 4A) by seeding the cells for 24 h in the different media and then treating them for 8 h with those ROS‐inducing agents. This approach decreased cellular viability more intensively in cells grown without cysteine/cystine. Indeed, 50 µm of tBH selectively induced cell death in 30–50% of cells but did not affect the viability of the cells grown in full medium. Likewise, 100 µm of H_2_O_2_ had the same deleterious effect. That ROS‐induced cell death could be partially reversed by adding trolox or GSH (Fig. 4B). Traditionally, BSO has been the drug of choice to target GSH synthesis and increase the sensitivity of cancer cells to oxidative stress; however, that approach has not been effective in some cases [40], including gliomas as a single agent [46]. We observed similar results for the BT142 cell line, in which BSO as a single agent did not reduce cellular viability (Fig. S5A), and the effect was much lower than that achieved by our approach. Similarly, nutrient limitation had a more intense effect on TS603 and NCH1681 cells because, unlike BSO, nutrient deprivation also affects the cells’ ability to regenerate GSH through glutathione reductase activity [47].

To test the effect of cysteine/cystine deprivation on survival, we conducted an *in vivo* experiment in which we injected mice intracranially with NCH1681 cells. Twelve days later, we randomized the mice into two groups and provided a cysteine/cystine‐free or a control diet the following day. We observed an increase in the mean survival of 26 days for those mice subjected to the cysteine/cystine‐free diet (Fig. 4C), although this difference did not attain statistical significance. We then monitored the plasma levels of the main metabolites related to cysteine, as potential biomarkers of treatment response at 16, 35, 44, and 55 days after intracranial injection. For each time point, metabolite levels were normalized to those computed in the control group. GSH and cystine levels in plasma were significantly lower in the cysteine/cystine‐free diet group 23 days after initiating the nutritional intervention (Fig. 4D). We also performed a ^13^C‐tracing experiment *in vivo* at the study end point by injecting [^13^C‐U]‐glutamine into the tail vein and tracking the incorporation of this metabolite into glutamate, glutamine, and GSH in order to evaluate the ability of tumor cells to synthetize GSH *de novo*. We observed active *de novo* synthesis of GSH in the cysteine/cystine‐free diet group, similar to that detected in the control group (Fig. 4E). Moreover, levels of ^13^C‐labeled m+5 glutamine were higher in the tumor region for both cohorts, as previously reported [48], and lower glutamate m+5 levels correlated more strongly with the cysteine/cystine‐free diet. GSH m+5 levels were higher than those detected in the respective contralateral regions, in accordance with a higher need for antioxidant capacity in tumor cells [49, 50]. Levels of ^13^C‐labeled GSH in tumor tissue under both diets were consistent with the recovery of circulating levels of cystine in plasma (Fig. 4D), indicating a systemic response to the nutritional intervention. Additional metabolites related to the cysteine pathway but diverted from the synthesis of GSH, such as taurine and hypotaurine, showed a different trend than those of cystine and GSH; that is, their levels diminished over time under the cysteine/cystine‐free diet (Fig. S5C). This temporary reduction in the main antioxidant source translated to nonsignificant enhancement of oxidative stress in the tumor tissue of mice given the cysteine/cystine‐free diet (Fig. 4F).

## 4 Discussion

We have shown how dietary limitation of the nonessential amino acid cysteine has an antiproliferative effect and can reshape the metabolic landscape of cancer cells. Although the deprivation of cysteine was recently shown to have a deleterious effect on tumor growth [51], the metabolic consequences triggered by this situation were unexplored. Here, we provided a broad description of the perturbations occurring in the metabolic pathways of glioma cells, highlighting the absence of an active TS pathway and offering a supplementary window of intervention during which to reduce the tumor’s ability to fight oxidative stress. It has been reported that limiting the exogenous contribution of cysteine is a potential treatment for prostate, pancreas, and breast cancers [52, 53], renal cell carcinomas [54], and leukemia [55].

Homocysteine can be remethylated yielding methionine, or it can be launched through the TS pathway. Recently, a disconnection between both routes was reported in cancer because the cells are unable to modulate the methylating enzyme [51], glycine *N*‐methyltransferase, which was resolved by ectopic overexpression of this enzyme. The methylation capacity of the glioma cell lines utilized in our study did not change a result of cysteine‐limited conditions (Fig. 3G). Rather, the constraint lies in the ability of the cells to divert the methionine cycle and generate downstream metabolites rather than upregulate enzymes involved in this route, because addition of the substrates for CBS and CTH recovered the cells’ growth and viability (Fig. 1E). Interestingly, a recent investigation revealed that the TS pathway can be also targeted in IDH1 mutant gliomas by unbalancing the antioxidant homeostasis of the tumor [56].

We detected incorporation of the ^13^C label from cysteine/cystine to cystathionine as well as overexpression of CTH after cysteine/cystine deprivation; thus, this result might suggest that cystathionine can act as a reservoir of cysteine. Then, in nutrient‐limited conditions, cystathionine can be broken down to yield cysteine. The disconnection between the TS pathway and the methionine cycle was reported recently in similar experiments that found that after addition of either cystathionine or homocysteine, both proliferation and recovery were restored, but not when cells were supplemented with methionine in a cysteine‐deficient medium [57]. The authors assign this effect to the diversion of methionine via SAM toward polyamine synthesis, a route that is upregulated is several cancers [58, 59]; indeed, targeting of polyamine synthesis and uptake in other brain tumors, that is, diffuse intrinsic pontine glioma has been recently proposed as a potential therapeutic alternative [60].

It is not known why some cancers can upregulate the TS pathway and do not rely on the uptake of cystine and cysteine [18]. Cysteine/cystine deprivation caused a 10‐fold reduction in GSH levels in our cell lines, thereby hypersensitizing glioma cells to ROS and revealing a high dependence on the exogenous supply of this amino acid. A recent investigation also showed how tumors that have large antioxidant capacity require an exogenous supply of nonessential amino acids for proliferation [61]. Targeting GSH synthesis has been a recurrent therapeutic strategy [52], mainly to sensitize tumor cells in combinatorial therapies [62, 63]. That approach has also been tested in IDH1‐mutant cancers [46], although in the present work, we observed how IDH1‐wild‐type gliomas were as sensitive as mutant gliomas to cysteine/cystine deprivation and that inhibiting IDH1‐mutant activity did not affect the response of those cells to this approach.

Tumor cells display enhanced oxidative stress, which is compensated for by a larger antioxidant capacity [13, 50]. Therefore, reducing the ability of tumor cells to fight against ROS can be an efficient strategy for treating cancer. From a pharmacologic point of view, brain tumors are challenging because of the selective permeability of the blood–brain barrier. Through our strategy, we could target the antioxidant capacity of tumor cells without using any drug therapy. Herein, we show that a cysteine‐deprivation diet creates a window of therapeutic opportunity by decreasing GSH and its precursor (cystine/cysteine) at the systemic level (Fig. 4D and Fig. S5C), which could then be utilized in combination with a ROS‐inducing agent. Although the plasma levels of these metabolites (GSH and cystine) were recovered afterward, those of hypotaurine and taurine (Fig. S5C), which are generated through the cysteine dioxygenase pathway, were not recovered. Nevertheless, homocysteine and methionine levels did not change significantly due to the diet (Fig. S5C). The selective recovery of metabolites related to the antioxidant response might indicate an enhanced demand for ROS‐buffering agents due to the increased oxidative stress generated by the diet. Nutritional interventions have been reported recently to be efficient strategies for tumor treatment in animal models [6], including a cysteine‐limitation strategy in a model of pancreatic cancer [64]. Our approach can be used clinically when it is necessary to limit the antioxidant capacity to enhance the effect of a secondary drug in a combinatorial treatment or when the resistance mechanism to a treatment depends on the ability of the cells to upregulate GSH metabolism.

## 5 Conclusions

In this study, we demonstrate that glioma cells rely on cysteine/cystine from the microenvironment for glutathione synthesis needed to fight ROS. This dependency is due to the inability of diverting methionine‐derived metabolites toward cysteine synthesis via the TS pathway. We show that via nutritional intervention alone, we can extend the survival of a mouse model of glioma, suggesting a time window for this type of intervention clinically.

### 5.1 Limitations of the study

Our investigation focused on IDH1‐mutant gliomas because they have been traditionally described as being more vulnerable to oxidative stress. However, this specificity should be further investigated by including IDH1‐wild‐type cell lines and a mouse model of IDH1‐wild‐type glioma in order to provide additional evidence for our findings. The *in vivo* experiment correlated the results from time series analysis of plasma with those obtained from tissue at end point; therefore, a longitudinal analysis of tumor tissue after nutritional intervention would add more value to our interpretation. This approach would require GSH to be monitored in tissue through magnetic resonance spectroscopy or by euthanizing the animals at different time points to extract the metabolites for analysis.

## Conflict of interest

The authors declare no conflict of interest.

## Author contributions

VR‐R and ML conceptualized the research and designed the experiments; VR‐R, TD, AL, JJ, LZ, and AD‐E performed experiments; TK and MZ assisted with animal work; CCH‐M provided cell lines; VR‐R, ML, KC, and MRG wrote the manuscript.

### Peer Review

The peer review history for this article is available at https://publons.com/publon/10.1002/1878‐0261.13148.

## Supporting information


**Fig. S1.** (A) DNA methylation‐based tumor classification and copy number variation plot of glioma cell lines including the classification scores arising from their methylome profile. NCH1681 cell lines was matched to high grade IDH^mut^ astrocytoma with a calibrated score of 0.94 while BT142 with a calibrated score of 0.99. TS603 was not matched to high grade astrocytoma. Copy number variation shows loss of 1p and 19q and with the presence of IDH1^mut^, this cell line is classified as a IDH1^mut^ oligodendroglioma 1p/19q co‐deleted. (B) Detection of IDH1 mutation in glioma cell lines by sequencing analysis. (C) Western blot of IDH1 mutant enzyme. (D) LC‐MS quantification of D2HG (mean ± SD for n = 3; **, *p* < 0.01; ***, *p* < 0.005 from a one‐way ANOVA followed by Tukey’s multiple comparison test) including a wild type glioma cell line (GSC923) as negative control.
**Fig. S2.** (A) Sphere formation of glioma cell lines after 96 hours growing in media with/out cysteine/cystine. Representative pictures are shown. (B) Western blots of glutamate cysteine ligase modulatory subunit (GCLM) for 3 IDH1 mutant glioma cell lines grown in media without cysteine/cystine and treated with 0.1 mM cystathionine or homocysteine. (C) Normalized intensity (n = 3, data displayed as mean ± SD; *, *p* < 0.05; **, *p* < 0.005, from a t‐test performed for each cell line) of the proteins bands relative to α‐tubulin expression from (D) western blots of p‐GCN2 and p‐eIF2α for 3 IDH1 mutant glioma cell lines grown in media without cysteine/cystine. (E) DCFDA intensity plots as marker of ROS in glioma cell lines under cysteine/cystine‐deprivation and treated with 0.25 mM Trolox including the relative quantification of positive‐stained cells (mean ± SEM for n = 3; *, *p* < 0.05; **, *p* < 0.005 from a one‐way ANOVA followed by Tukey’s multiple comparison test). (F) Viability of glioma cell lines in cysteine/cystine‐lacking media supplemented with either GSH or GSH‐ethyl ester (GSH‐E) (mean ± SD for n = 3; *, *p*  <  0.05 versus control by two‐tailed Student’s *t*‐test) and (G) quantification of intracellular GSH (mean ± SD for n = 3; *, *p* <  0.05; **, *p* < 0.005; ***, *p* < 0.001 from a two‐way ANOVA followed by Tukey’s multiple comparison test). (H) Viability of glioma cell lines after 96 hours in media lacking cysteine and cystine and in the same media but treated with 2 µM ferrostatin 1. (n = 3, bar plots displaying mean ± SD normalized to results from experiments performed in full media). (I) Quantification of the number of cells assigned to early apoptosis and apoptosis (n = 3, bar plots displaying mean ± SD normalized to results from experiments performed in full media) from the apoptosis detection assay diagrams at 96 hours and (J).
**Fig. S3.** (A) Relative intensity of the CBS and CTH bands normalized to β‐actin expression (n = 3, data displayed as mean ± SD; *, *p* < 0.05; **, *p* < 0.005, from a t‐test performed for each cell line) from the western blots of CBS and CTH for 3 IDH1 mutant glioma cell lines grown in media without cysteine/cystine. (B) Metabolic effect of propargylglycine (PAG) due to inhibition of the TS pathway. (C) Volcano plot displaying the Log_2_(FC) vs Log(FDR *p* value) for all the metabolites identified by LC‐MS. Metabolites highlighted in green (upregulated upon PAG treatment) or red (downregulated) have Log_2_(FC) > 1 or < −1 and an FDR *p* value < 0.05 (5 replicates per cell line and per condition), in addition to glutathione (GSH).
**Fig. S4.** Levels of (A) tetrahydrofolate (THF) and folate, (B) Relative quantification of the expression of MTHFD1 as the normalized intensity of its band to β‐actin (normalized intensities from n = 3, data displayed as mean ± SD; none of the comparisons attained statistical significance from a t‐test performed for each cell line) and (C) citrulline and arginine for the 3 cell lines in full and cysteine/cystine‐lacking media for 48 hours. Metabolite levels are computed from the unlabeled global profiling experiment (n = 5, bar plots displaying mean values ± SD, p‐values arising from a t‐test with Welch correction adjusted for multiple comparisons by the FDR method. **, *p* < 0.005; ***, *p* <  0.001).
**Fig. S5.** (A) Effect of BSO plus tBH on viability. Data are mean ± SD of viability values normalized to the control conditions (full medium), n = 3; *p < 0.05, **p < 0.005, ***p < 0.001, 2‐way ANOVA followed by Dunnet’s multiple comparison test for control vs. all). (B) Quantification of GSH levels after treatment with 250 µM BSO. (C) Normalized intensities of cysteine‐related metabolites in plasma collected from a mouse model of glioma and normalized to those computed for the control group (dotted line for reference values of the control group and metabolite levels for diet‐group displayed as mean ± SEM).

## Data Availability

Data and reagents generated during the study are available from the corresponding author (mioara.larion@nih.gov) upon reasonable request.
