# Supplementary material for: Cysteine is a limiting factor for glioma proliferation and survival
Source: Mol Oncol. 2022 Jan 6;16(9):1777–94. doi: 10.1002/1878-0261.13148 (PMC9067152; doi:10.1002/1878-0261.13148)
Supplement: Supplementary file 1 — Fig. S1. (A) DNA methylation‐based tumor classification and copy number variation plot of glioma cell lines including the classification scores arising from their methylome profile. NCH1681 cell lines was matched to high grade IDHmut astrocytoma with a calibrated score of 0.94 while BT142 with a calibrated score of 0.99. TS603 was not matched to high grade astrocytoma. Copy number variation shows loss of 1p and 19q and with the presence of IDH1mut, this cell line is classified as a IDH1mut oligodendroglioma 1p/19q co‐deleted. (B) Detection of IDH1 mutation in glioma cell lines by sequencing analysis. (C) Western blot of IDH1 mutant enzyme. (D) LC‐MS quantification of D2HG (mean ± SD for n = 3; **, p < 0.01; ***, p < 0.005 from a one‐way ANOVA followed by Tukey’s multiple comparison test) including a wild type glioma cell line (GSC923) as negative control. Fig. S2. (A) Sphere formation of glioma cell lines after 96 hours growing in media with/out cysteine/cystine. Representative pictures are shown. (B) Western blots of glutamate cysteine ligase modulatory subunit (GCLM) for 3 IDH1 mutant glioma cell lines grown in media without cysteine/cystine and treated with 0.1 mM cystathionine or homocysteine. (C) Normalized intensity (n = 3, data displayed as mean ± SD; *, p < 0.05; **, p < 0.005, from a t‐test performed for each cell line) of the proteins bands relative to α‐tubulin expression from (D) western blots of p‐GCN2 and p‐eIF2α for 3 IDH1 mutant glioma cell lines grown in media without cysteine/cystine. (E) DCFDA intensity plots as marker of ROS in glioma cell lines under cysteine/cystine‐deprivation and treated with 0.25 mM Trolox including the relative quantification of positive‐stained cells (mean ± SEM for n = 3; *, p < 0.05; **, p < 0.005 from a one‐way ANOVA followed by Tukey’s multiple comparison test). (F) Viability of glioma cell lines in cysteine/cystine‐lacking media supplemented with either GSH or GSH‐ethyl ester (GSH‐E) (mean ± SD for n = 3; *, p < 0.05 [file MOL2-16-1777-s001.docx]

**SUPPLEMENTARY INFORMATION**

**SUPPLEMENTARY FIGURES**

**
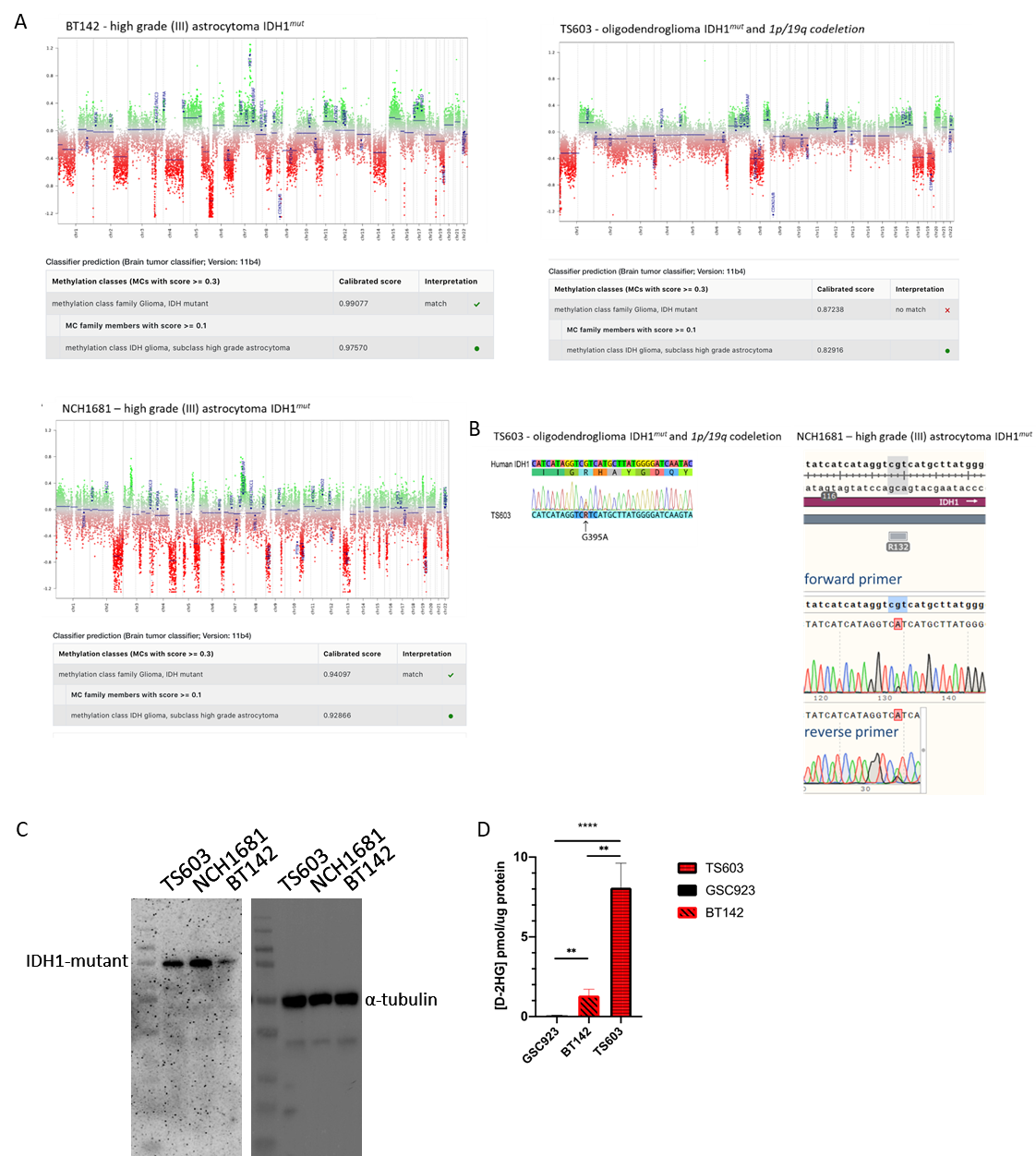
**

**Supplementary Figure 1:** (A) DNA methylation-based tumor classification and copy number variation plot of glioma cell lines including the classification scores arising from their methylome profile. NCH1681 cell lines was matched to high grade IDH^mut^ astrocytoma with a calibrated score of 0.94 while BT142 with a calibrated score of 0.99. TS603 was not matched to high grade astrocytoma. Copy number variation shows loss of 1p and 19q and with the presence of IDH1^mut^, this cell line is classified as a IDH1^mut^ oligodendroglioma 1p/19q co-deleted. (B) Detection of IDH1 mutation in glioma cell lines by sequencing analysis. (C) Western blot of IDH1 mutant enzyme. (D) LC-MS quantification of D2HG (mean ± SD for n=3;  **, *p*< 0.01; ***, *p*<0.005 from a one-way ANOVA followed by Tukey’s multiple comparison test) including a wild type glioma cell line (GSC923) as negative control.

**
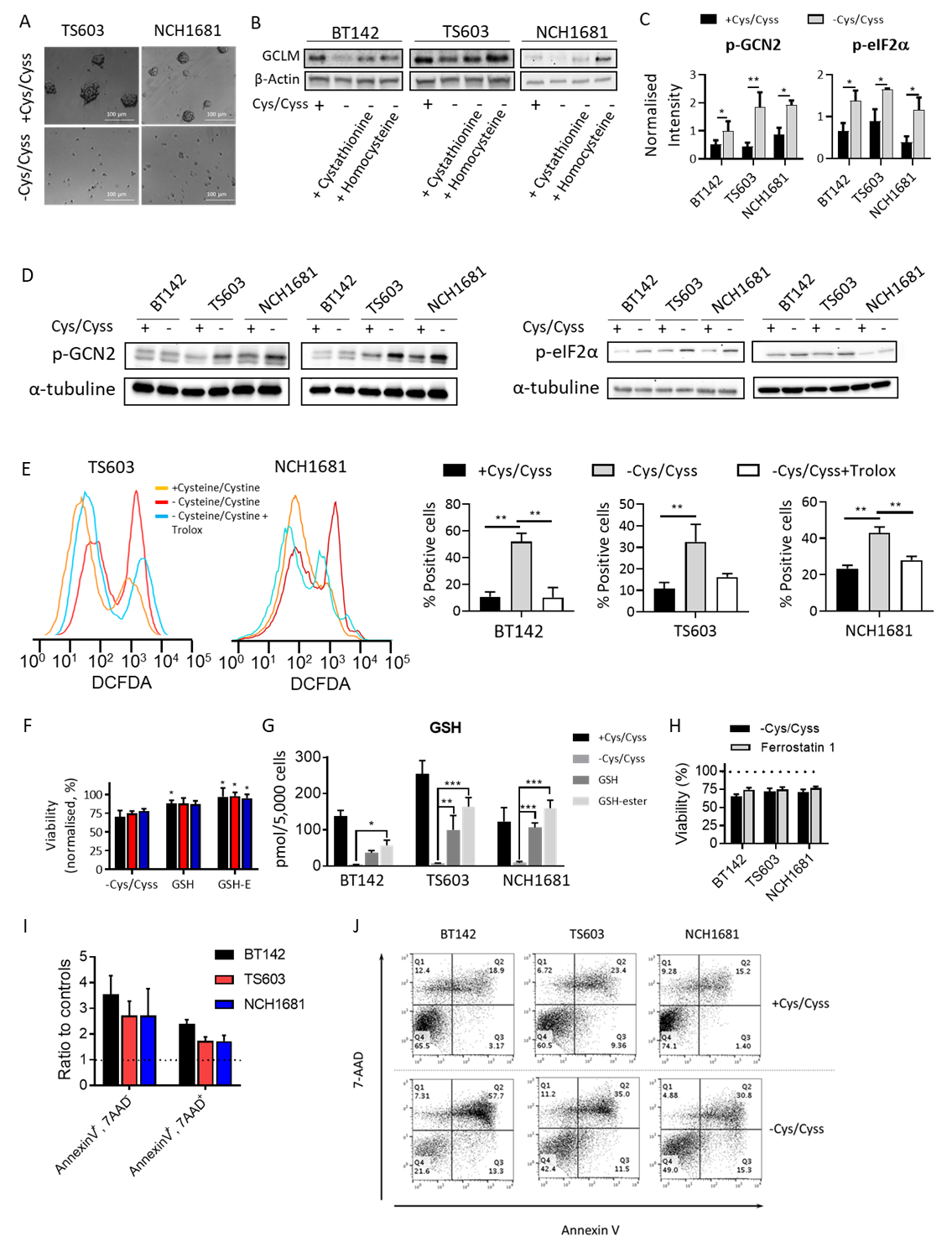
**

**Supplementary Figure 2:** (A) Sphere formation of glioma cell lines after 96 hours growing in media with/out cysteine/cystine. Representative pictures are shown. (B) Western blots of glutamate cysteine ligase modulatory subunit (GCLM) for 3 IDH1 mutant glioma cell lines grown in media without cysteine/cystine and treated with 0.1 mM cystathionine or homocysteine. (C) Normalized intensity (n=3, data displayed as mean ± SD; *, *p* < 0.05; **, *p* < 0.005, from a t-test performed for each cell line) of the proteins bands relative to α-tubulin expression from (D) western blots of p-GCN2 and p-eIF2α for 3 IDH1 mutant glioma cell lines grown in media without cysteine/cystine. (E) DCFDA intensity plots as marker of ROS in glioma cell lines under cysteine/cystine-deprivation and treated with 0.25 mM Trolox including the relative quantification of positive-stained cells (mean ± SEM for n=3;  *, *p*< 0.05; **, *p*<0.005 from a one-way ANOVA followed by Tukey’s multiple comparison test). (F) Viability of glioma cell lines in cysteine/cystine-lacking media supplemented with either GSH or GSH-ethyl ester (GSH-E) (mean ± SD for n =3; *, *p* < 0.05 versus control by two-tailed Student’s *t*-test) and (G) quantification of intracellular GSH (mean ± SD for n=3; *, *p*< 0.05; **, *p*<0.005; ***, *p*<0.001 from a two-way ANOVA followed by Tukey’s multiple comparison test). (H) Viability of glioma cell lines after 96 hours in media lacking cysteine and cystine and in the same media but treated with 2 µM ferrostatin 1. (n=3, bar plots displaying mean ± SD normalized to results from experiments performed in full media). (I) Quantification of the number of cells assigned to early apoptosis and apoptosis (n=3, bar plots displaying mean ± SD normalized to results from experiments performed in full media) from the apoptosis detection assay diagrams at 96 hours and (J).

**
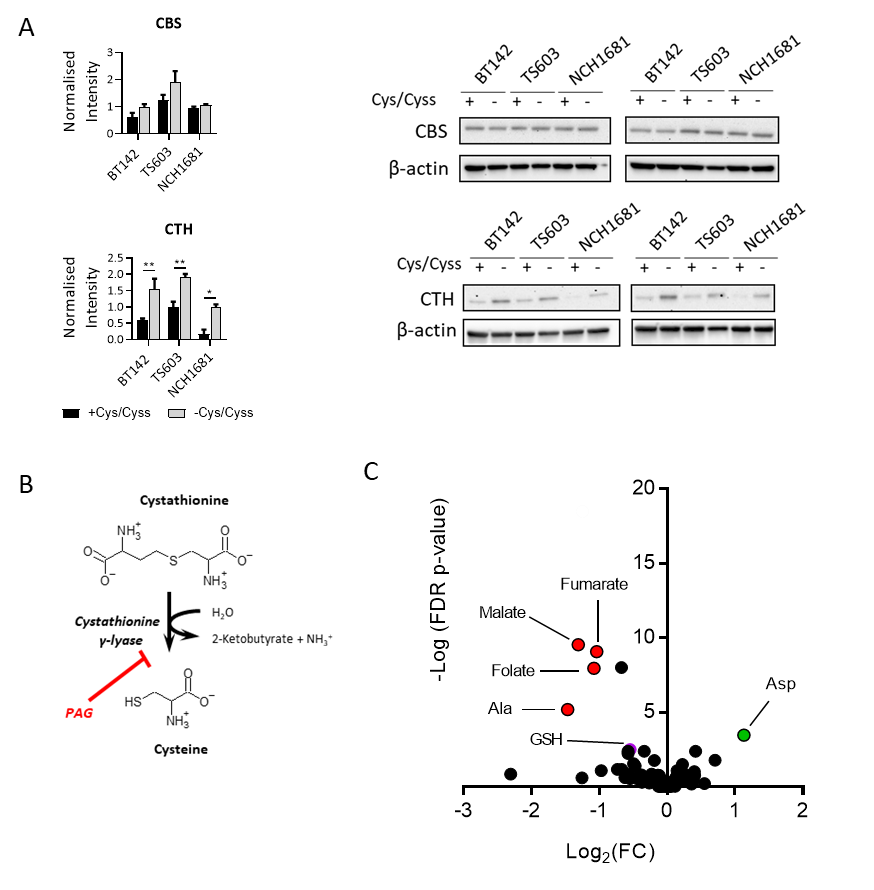
**

**Supplementary Figure 3**: (A) Relative intensity of the CBS and CTH bands normalized to β-actin expression (n=3, data displayed as mean ± SD; *, *p* < 0.05; **, *p* < 0.005, from a t-test performed for each cell line) from the western blots of CBS and CTH for 3 IDH1 mutant glioma cell lines grown in media without cysteine/cystine. (B) Metabolic effect of propargylglycine (PAG) due to inhibition of the TS pathway. (C) Volcano plot displaying the Log_2_(FC) vs Log(FDR *p* value) for all the metabolites identified by LC-MS. Metabolites highlighted in green (upregulated upon PAG treatment) or red (downregulated) have Log_2_(FC) > 1 or < -1 and an FDR *p* value<0.05 (5 replicates per cell line and per condition), in addition to glutathione (GSH).


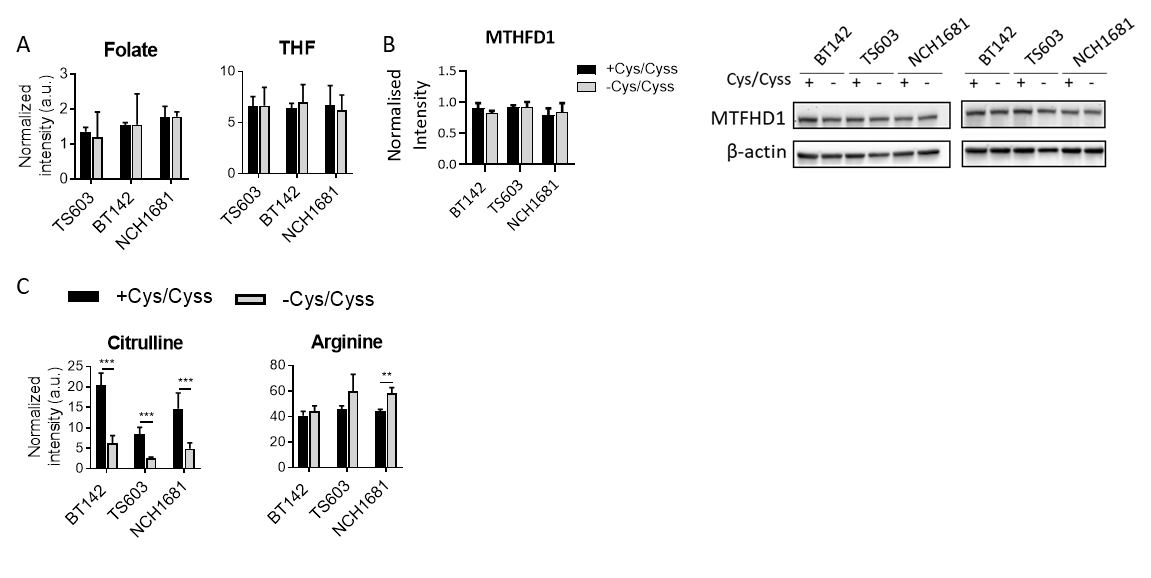


**Supplementary Figure 4**: Levels of (A) tetrahydrofolate (THF) and folate, (B) Relative quantification of the expression of MTHFD1 as the normalized intensity of its band to β-actin (normalized intensities from n=3, data displayed as mean ± SD; none of the comparisons attained statistical significance from a t-test performed for each cell line) and (C) citrulline and arginine for the 3 cell lines in full and cysteine/cystine-lacking media for 48 hours. Metabolite levels are computed from the unlabeled global profiling experiment (n=5, bar plots displaying mean values ± SD, p-values arising from a t-test with Welch correction adjusted for multiple comparisons by the FDR method. **, *p*<0.005; ***, *p*<0.001).

**
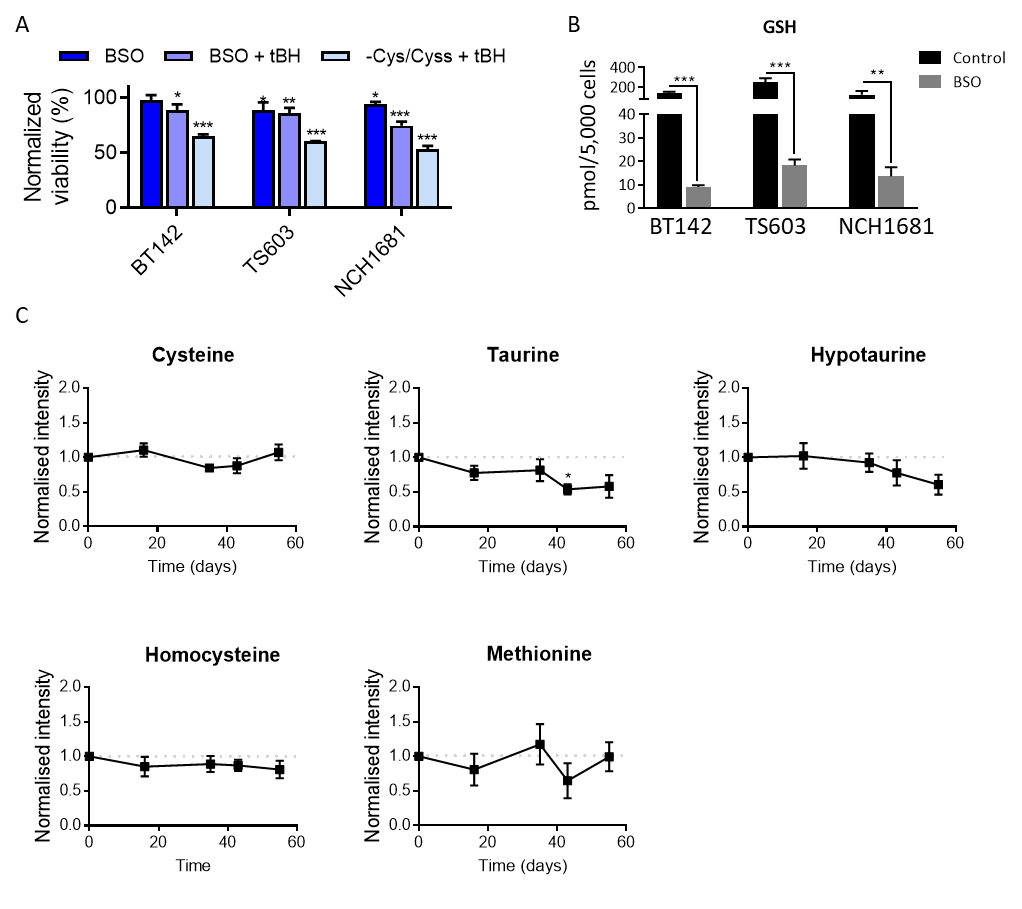
**

**Supplementary Figure 5**: (A) Effect of BSO plus tBH on viability. Data are mean ± SD of viability values normalized to the control conditions (full medium), n = 3; **p*< 0.05, ***p*< 0.005, ****p*< 0.001, 2-way ANOVA followed by Dunnet’s multiple comparison test for control vs. all). (B) Quantification of GSH levels after treatment with 250 µM BSO. (C) Normalized intensities of cysteine-related metabolites in plasma collected from a mouse model of glioma and normalized to those computed for the control group (dotted line for reference values of the control group and metabolite levels for diet-group displayed as mean ± SEM).
